# Binary Biomass-Based Electrolyte Films for High-Performance All-Solid-State Supercapacitor

**DOI:** 10.3390/polym16192772

**Published:** 2024-09-30

**Authors:** Rui Lou, Guocheng Zhang, Taoyuan Niu, Long He, Ying Su, Guodong Wei

**Affiliations:** 1 College of Mechanical and Electrical Engineering, Shaanxi University of Science and Technology, Xi’an 710021, China; 230512146@sust.edu.cn (G.Z.); niuty2007@163.com (T.N.); helong@sust.edu.cn (L.H.); 2 Materials Institute of Atomic and Molecular Science, Shaanxi University of Science and Technology, Xi’an 710021, China; suying8010@163.com (Y.S.); wgd588@163.com (G.W.)

**Keywords:** lignin nanoparticles, sodium alginate, solid electrolyte, supercapacitor, electrochemical

## Abstract

Solid-state electrolytes have received widespread attention for solving the problem of the leakage of liquid electrolytes and effectively improving the overall performance of supercapacitors. However, the electrochemical performance and environmental friendliness of solid-state electrolytes still need to be further improved. Here, a binary biomass-based solid electrolyte film (LSE) was successfully synthesized through the incorporation of lignin nanoparticles (LNPs) with sodium alginate (SA). The impact of the mass ratio of SA to LNPs on the microstructure, porosity, electrolyte absorption capacity, ionic conductivity, and electrochemical properties of the LSE was thoroughly investigated. The results indicated that as the proportion of SA increased from 5% to 15% of LNPs, the pore structure of the LSE became increasingly uniform and abundant. Consequently, enhancements were observed in porosity, liquid absorption capacity, ionic conductivity, and overall electrochemical performance. Notably, at an SA amount of 15% of LNPs, the ionic conductivity of the resultant LSE-15 was recorded at 14.10 mS cm^−1^, with the porosity and liquid absorption capacity reaching 58.4% and 308%, respectively. LSE-15 was employed as a solid electrolyte, while LNP-based carbon aerogel (LCA) served as the two electrodes in the construction of a symmetric all-solid-state supercapacitor (SSC). The SSC device demonstrated exceptional electrochemical storage capacity, achieving a specific capacitance of 197 F g^−1^ at 0.5 A g^−1^, along with a maximum energy and power density of 27.33 W h kg^−1^ and 4998 W kg^−1^, respectively. Furthermore, the SSC device exhibited highly stable electrochemical performance under extreme conditions, including compression, bending, and both series and parallel connections. Therefore, the development and application of binary biomass-based solid electrolyte films in supercapacitors represent a promising strategy for harnessing high-value biomass resources in the field of energy storage.

## 1. Introduction

Supercapacitors (SCs) are recognized as one of the most promising types of energy storage devices due to their high power density, long cycle life, and rapid charging and discharging capabilities, as well as their economic and environmental benefits [1,2,3]. However, the majority of commercially available SCs employ liquid electrolytes, which are susceptible to leakage, have a limited operational lifespan, and pose safety concerns, thereby impeding their further development [4,5]. Particularly under conditions of high intensity or harsh environments, the leakage of liquid electrolytes significantly increases the risk of severe incidents, such as short circuits and fires. In contrast, solid-state electrolytes effectively mitigate the issue of electrolyte leakage, enhancing the safety and reliability of the devices. Furthermore, solid-state electrolytes exhibit superior chemical stability and environmental adaptability, which contribute to improving the overall performance and service life of supercapacitors. Therefore, the advancement of novel solid-state electrolytes, particularly those that meet various criteria such as safety, high efficiency, and environmental sustainability, has emerged as a crucial approach to address these challenges.

Solid-state electrolytes can be categorized into three primary types based on their composition: polymer electrolytes, inorganic electrolytes, and composite electrolytes [6,7]. In recent years, composite solid-state electrolytes have become a focal point of research within the domain of solid-state electrolytes, primarily due to their superior characteristics that combine the benefits of both polymer and inorganic solid-state electrolytes. These advantages include high ionic conductivity, a wide operating temperature range, and favorable mechanical properties [8]. The incorporation of biomass-based materials has shown the enhanced performance of solid electrolytes, along with the most benefits of environmental sustainability, abundance, and low cost. Natural polymers, such as cellulose, lignin, chitosan, and sodium alginate, contain a large amount of hydroxyl groups and/or carboxyl groups, which can form hydrogen bonds with other substances, which are beneficial to ion conduction [1]. For instance, Pu et al. [9] utilized delignified wood as a raw material to fabricate a composite solid-state electrolyte that exhibited high ionic conductivity (29 mS cm^−1^ at room temperature) through a vacuum infusion process, incorporating polyethylene glycol and 2,2-hydroxymethyl propionic acid as the filler polymer matrix in varying ratios. The specific capacitance of the solid-state supercapacitor (SSC) constructed from this electrolyte reached 157.7 F g^−1^ at 0.3 A g^−1^. Similarly, Sun et al. [10] developed a composite solid-state electrolyte membrane via solution casting, employing Li_6.75_La_3_Zr_1.75_Nb_0.25_O_12_ nanowires, which possess a one-dimensional garnet structure, along with poly(methyl methacrylate) and lithium perchlorate. This composite solid-state electrolyte demonstrated an ionic conductivity of 2.2 × 10^−2^ mS cm^−1^ at room temperature. Additionally, Park et al. [11] synthesized a lignin-based hydrogel electrolyte through a chemical crosslinking method, which exhibited an ionic conductivity of 10.35 mS cm^−1^ at room temperature. When integrated with electrospun lignin/polyacrylonitrile nanofiber electrodes to form a flexible SC device, it achieved a specific capacitance of 129.23 F g^−1^ at 0.5 A g^−1^, maintaining a capacitance retention rate of 95% over 10,000 cycles. Nevertheless, the current research on biomass-based composite solid-state electrolytes remains in its infant stage, necessitating further enhancement and optimization of their performance through more comprehensive investigations.

Herein, a binary biomass composite system was developed utilizing lignin nanoparticles (LNPs) derived from wheat straw, which were compounded with sodium alginate (SA). The abundant methoxy and phenolic hydroxyl groups present in lignin were employed to crosslink with polyethylene glycol diglycidyl ether (PEGDGE). Additionally, the pore structure was optimized with SA, which is rich in hydrophilic groups. As a result, a binary LNP/SA-based solid-state electrolyte film (LSE) was successfully fabricated. This LSE was subsequently integrated with the LNP-based carbon aerogel (LCA) previously developed by our research group to create a symmetric all-solid-state supercapacitor (SSC). This assembly aimed to elucidate the energy storage capabilities of the fully biomass-based SC device.

## 2. Materials and Methods

### 2.1. Materials

Lignin nanoparticles (LNPs) extracted from wheat straw and LNP-based carbon aerogels (LCAs) utilized in this study were prepared following previously established procedures [12]. Sodium alginate (SA) was obtained from Shanghai Macklin Bio-Chem Technology Co., Ltd. (Shanghai, China). Poly (ethylene glycol) diglycidyl ether (PEGDGE, Mw = 500 mmol g^−1^) was procured from Shanghai Aladdin Bio-Chem Technology Co., Ltd. (Shanghai, China). Potassium hydroxide (KOH, 85%) was purchased from Tianjin Damao Chemical Reagent Factory (Tianjin, China). All reagents employed were analytical grade and used as received without further purification.

### 2.2. Preparation of the LSE

A specific quantity of SA and 0.3 g of LNPs were introduced into 1 mL of a 3.3 M KOH solution. The resulting mixture was subjected to magnetic stirring for a duration of 12 h to ensure complete dissolution. Following this, 0.18 g of PEGDGE was added to the mixture and stirred magnetically for 30 min. The homogenously mixed solution was subsequently transferred into a clean and dry mold and incubated at 40 °C for a crosslinking reaction that lasted 12 h, resulting in the successful formation of the LNP/SA-based solid-state electrolyte film (LSE). The mass fraction of composite SA was varied at 5%, 10%, 15%, and 20% relative to the mass of LNPs, with the corresponding prepared LSEs designated as LSE-5, LSE-10, LSE-15, and LSE-20, respectively.

### 2.3. The Property Tests of the LSE

The assessment of the porosity of a fresh LSE was performed as specified below: The LSE specimen was immersed in *n*-butyl alcohol for a duration of 6 h, after which any excess liquid on the surface was removed [13]. The porosity was calculated using the formula provided in Equation (1).

(1) $$Porosity=\frac{W_{a}-W_{b}}{\rho V}\times 100\%$$

where *W_b_* (g) is the weight of the initial LSE, *W_a_* (g) is the weight of a soaked LSE in *n*-butyl alcohol, *ρ* (g cm^−3^) is the density of *n*-butyl alcohol, and *V* (cm^3^) is the LSE volume.

The liquid/electrolyte absorption capacity (*η_a_*) of the fresh LSE was evaluated by immersing the LSE in a 3.3 M KOH solution for 2 h and determined using Equation (2) [13].

(2) $$\eta_{a}=\frac{W_{2}-W_{1}}{W_{1}}\times 100\%$$

where *W*_1_ (g) is the weight of the initial LSE, and *W*_2_ (g) is the weight of the soaked LSE.

The ionic conductivity of the LSE was assessed using the electrochemical impedance spectroscopy (EIS) method [14]. The LSE specimen was placed between two parallel stainless steel sheets and configured into a CR2032-type coin cell. The frequency range for measurement was set from 0.01 Hz to 100 kHz at ambient temperature. The ionic conductivity (*δ*, mS cm^−1^) was determined by applying Equation (3).

(3) $$\delta =\frac{1000D}{RA}$$

where *D* (cm) is the thickness of the swollen LSE, *R* (Ω) is the bulk resistance in EIS, and *A* (cm^2^) is the contact area of the swollen LSE with stainless steel sheets.

### 2.4. Characterizations

The surface morphology of dry LSE samples was conducted on a scanning electron microscope (SEM, FEI Verios 460, Hillsboro, OR, USA). The dry films were attached onto the specimen stage, then coated with an Au film as a sputter coater, after which the coated samples were examined at an accelerating voltage of 10 kV. The elemental composition of the dry LSE sample was analyzed by X-ray photoelectron spectroscopy (XPS, Kratos, AXIS Supra, Manchester, UK).

### 2.5. Assembly of SSC Devices

LNP-based carbon aerogel (LCA) was utilized as the active material in the working electrodes, which were prepared following established procedures [15].

The working electrodes were fabricated by blending LCA, acetylene black, and polytetrafluoroethylene (PTFE) binder in ethanol at a mass ratio of 8:1:1 to create a slurry. This slurry was then applied onto a nickel foam (10 mm × 20 mm) and dried at 80 °C for 12 h. A sandwich structure was fabricated by compressing the LSE with two working electrodes of equal mass, followed by encapsulation with PET film to obtain the SSC device.

### 2.6. Electrochemical Measurements

The electrochemical performances of the as-prepared SSC devices were determined on an electrochemical workstation (DH 7000C, Jiangsu Donghua Analytical Instruments CO., Ltd., Taizhou, China), covering the cyclic voltammetry (CV), galvanostatic charge/discharge (GCD), and EIS measurements. The frequency range of EIS was detected from 100 kHz to 0.01 Hz at an open circuit potential. The respective specific capacitance (*C*, F g^−1^), energy density (*E*, W h kg^−1)^, and power density (*P*, W kg^−1^) of the SSC devices were calculated by the following Equations (4)–(6).

(4) $$C=\frac{2I\times \Delta t}{m\times \Delta V}$$

(5) $$E=\frac{C\times \Delta V^{2}}{2\times 3.6}$$

(6) $$P=\frac{3600E}{\Delta t}$$

where Δ*t* (s) is the discharge time, *I* (A) is the discharge current, *m* (g) is the total loading mass of the electrodes, and Δ*V* (V) is the potential window.

## 3. Results

**Figure 1:**
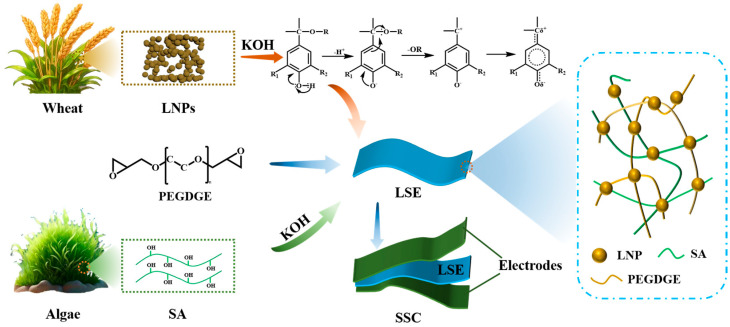
Schematic representation of the LSE preparation process.

The schematic representation for the fabrication of the LSE is illustrated in Figure 1. Initially, LNPs derived from wheat straw through deep eutectic solvent (DES) extraction can be effectively dissolved in a KOH solution. Under alkaline conditions, LNPs become fully activated, resulting in the formation of a methylene quinone structure (–C_4_–O^−^), which enhances the surface reactivity of lignin. Notably, the presence of negatively charged oxygen ions serves as a potent activating group, which facilitates the activation of the benzene ring, thereby improving the hydrophilicity of the lignin molecule and increasing its solubility [16]. Furthermore, the oxygen atom within the epoxy group of poly (ethylene glycol) diglycidyl ether (PEGDGE) exhibits low reactivity, rendering it regioselective under strong alkaline conditions [11]. The C*_α_* position on the lignin structure acts as a site for nucleophilic attack [17] leading to further interaction with the epoxy group of PEGDGE and initiating a ring-opening reaction. Following the cleavage of the epoxy ether bond, LNPs engage in a molecular cross-linking reaction with PEGDGE, while SA contributes to the formation of a homogeneous 3D network structure within the reaction solution. This network is established through molecular chain entanglement and interactions, such as hydrogen bonding [18], which facilitate rapid ion transport by shortening the transport distance [19]. In the preparation process, LNPs serve as the polymeric molecular backbone, SA functions as the network-enhancing matrix, and PEGDGE acts as the cross-linking agent, collectively enabling the development of binary LNP/SA-based solid-state electrolyte films (LSE).

### 3.1. Structural Characterization of the LSE

**Figure 2:**
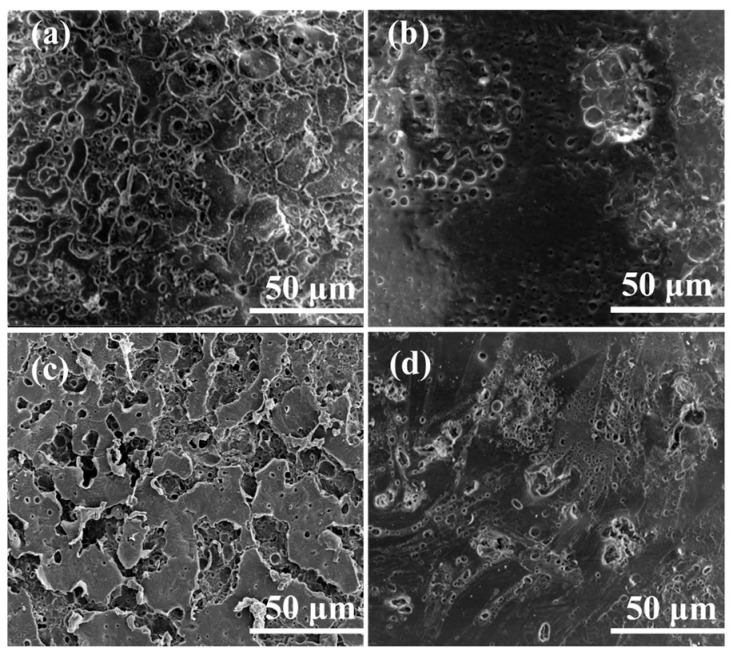
SEM images of (**a**–**c**) the LSE-5, LSE-10, LSE-15, and (**d**) LSE-20.

The SEM images of the LSE with varying concentrations of SA are presented in Figure 2. As the concentration of SA increased (Figure 2a–c), the number of pores within the LSE became more abundant and uniformly distributed, while the degree of cross-linking among the pores also intensified. This phenomenon can be attributed to the solubilization of hydroxyl-rich long-chain SA within the reaction system, which facilitates the intertwining of SA molecular chains and the establishment of a 3D network through intermolecular interactions, such as hydrogen bonding. Consequently, this leads to an increase in the flow resistance of the system [18]. However, when the SA concentration reaches 20% (Figure 2d), a decrease in pore size and a significant reduction in number of pore channels are observed. This reduction is ascribed to the excess SA, which elevates the viscosity of the reaction system and impedes the movement of the reaction components, thereby hindering the polymerization process.

### 3.2. Physicochemical Performance of the LSE

**Figure 3:**
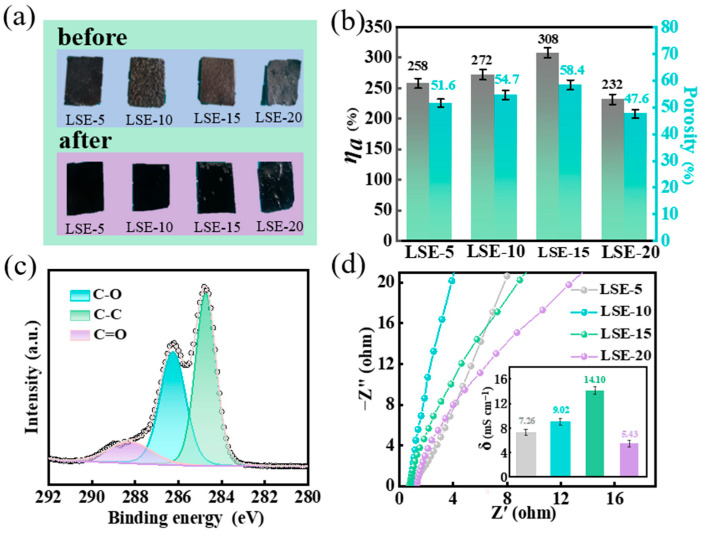
(**a**) Graphical comparisons of the LSE before and after immersion in a 3.3 M KOH solution for 4 h; (**b**) porosity and electrolyte absorption capacity (*η_a_*); (**c**) C 1s XPS spectra of LSE-15; (**d**) EIS curves and the ionic conductivity (*δ*) of the LSE.

Porosity and electrolyte absorption capacity are critical parameters for solid electrolyte films. The morphology of the films remains intact and exhibits favorable mechanical properties following infiltration with the electrolyte (Figure 3a). Additionally, the excellent swelling ability and infiltration characteristics of the LSE result in a modest increase in the film’s volume [17]. The porosity and electrolyte absorption capacity illustrated in Figure 3b reflect the swelling capacity of the LSE in the electrolyte solution. Notably, LSE-15 demonstrated the highest porosity at 58.4% and electrolyte absorption capacity of 308%. This observation is consistent with the more porous morphology of LSE-15, indicating that the 3D porous network structure facilitates enhanced charge carrier mobility and improved ionic conductivity [13].

As can be seen from Figure 3c, the C 1s spectra of LSE-15 predominantly exhibit peaks at 284.4, 286.1, and 288.8 eV. The peak at 284.4 eV is associated with C–C bonding, which is commonly found in LNPs and SA. The peak at 286.1 eV corresponds to C–O bonds, primarily arising from the substantial presence of alcohol hydroxyl (C–OH) and ether bonds (C–O–C) in LNPs and SA [20]. Conversely, the peak at 288.8 eV is attributed to the C=O bond, predominantly existing in the form of aldehyde groups (–CHO) in LNPs and ester groups (–COOR) in SA [21]. The hydroxyl groups and ether bonds exhibit hydrophilic properties, facilitating the formation of hydrogen bonds with water molecules, thereby enhancing the liquid absorption rate of the LSE. Additionally, the ester group functions as a cross-linking point within the spatial network of the LSE, contributing to the structural integrity of the LSE by preventing rupture or dissolution. Furthermore, an optimal content of ester groups induces a stretching conformation in the molecular chains of the LSE network, which can further enhance porosity and liquid absorption rates. However, an excessive concentration of ester groups may lead to increased network density, which is detrimental to the pore formation and solubility behaviors [22]. This observation aligns with the results of SEM, porosity assessments, and electrolyte absorption capacity analyses. As depicted in Figure 3d, the ionic conductivities of LSE-5, LSE-10, LSE-15, and LSE-20 were measured at 7.26, 9.02, 14.10, and 5.43 mS cm^−1^, respectively. It is evident that the ionic conductivity of the LSE reaches its peak at a 15% concentration of SA. This enhancement is primarily attributed to the provision of hydrophilic groups by SA within the solid electrolyte films, resulting in a more complex 3D crosslinked porous structure that facilitates faster ion transport rate as SA content increases. However, for LSE-20, an excessive amount of SA can lead to significantly increased viscosity of the solution, which restricts the size and number of pore channels. This limitation impedes the migration speed of ions during transport, ultimately resulting in reduced ionic conductivity.

### 3.3. Electrochemical Performance of SSC Devices

**Figure 4:**
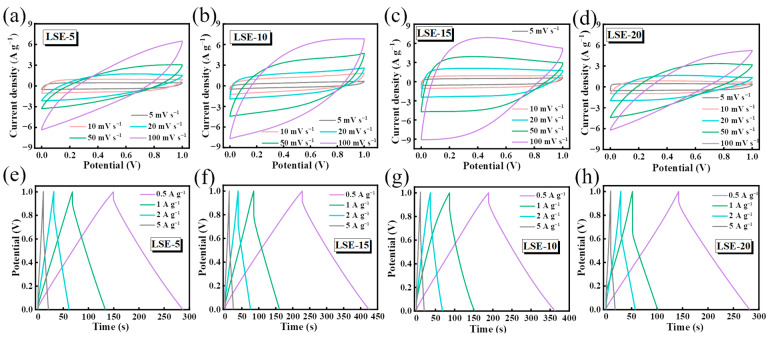
CV curves of (**a**) LSE-5, (**b**) LSE-10, (**c**) LSE-15, (**d**) LSE-20 at different scan rates; GCD curves of (**e**) LSE-5, (**f**) LSE-10, (**g**) LSE-15, (**h**) LSE-20 at different current densities.

Sustainable symmetrical SCC devices were fabricated utilizing the all LNP-based LCA as the electrodes and the LSE electrolyte. The CV curves of the SSC devices are presented in Figure 4a–d. The CV curves from LSE-15-based SSC demonstrate a regular symmetrical rectangular shape with a well-defined enclosed area. The absence of distinct redox peaks suggests favorable bilayer behavior and excellent charge/discharge reversibility [23]. Notably, even at elevated scanning rates of 100 mV s^−1^, the CV curves retain a rectangular-like form without significant distortion, indicating that the SSC exhibits commendable electrochemical performance and highly cyclic stability. However, the CV curves from LSE-5 and LSE-20-based SSC devices exhibited an oval-like shape with a smaller area, which may be attributed to the lower porosity and ionic conductivity of these electrolytes. The GCD curves at varying current densities, ranging from 0.5 to 5 A g^−1^, are illustrated in Figure 4e–h. The linear potential−time profiles, resembling an isosceles triangle, demonstrate that the all-LNP-based SSC device, composed of the LSE and LCA, possesses favorable fast charging and discharging behaviors, as well as bilayer behavior [24,25]. As the current density increases, the GCD curves continue to maintain the intrinsic isosceles triangle shape, further indicating that the SSC devices exhibit high stability and charge/discharge reversibility [26].

**Figure 5:**
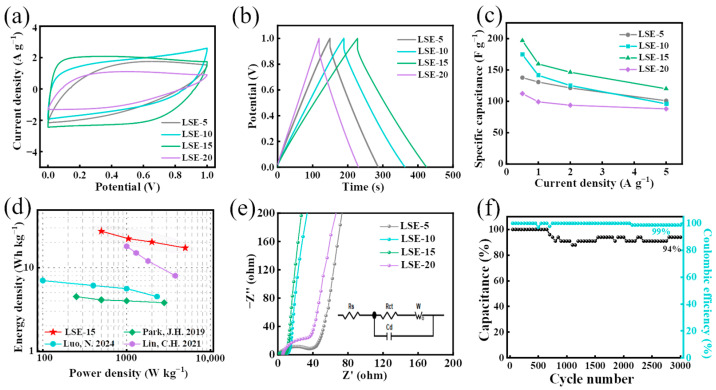
(**a**) CV curves of the SSC at 20 mV s^−1^; (**b**) GCD curves of the SSC at 0.5 A g^−1^; (**c**) specific capacitance; (**d**) Ragone plots [11,28,29]; (**e**) EIS curves; (**f**) long-term cycling stability and coulombic efficiency.

The CV curves of all LSE-based SSC devices obtained at a scan rate of 20 mV s^−1^ and their GCD curves recorded at a current density of 0.5 A g^−1^ are presented in Figure 5a,b, respectively. It is evident that the SSC devices constructed from LSE-15 exhibit the highest response current and the largest enclosed area under the CV curve (Figure 5a), indicating that it possesses the greatest specific capacitance. This superior performance can be attributed to the high electrolyte absorption, porosity, and ionic conductivity of LSE-15, which facilitate adequate ion transport and transfer during the charging/discharging processes of the SSC. A notable variation in discharge time for the SSC is observed in Figure 5b. The SSC assembled from LSE-15 demonstrates the longest discharge time, further corroborating its largest specific capacitance, which aligns with the findings from the CV curve analysis. The specific capacitances of the SSCs constructed from LSE-5, LSE-10, LSE-15, and LSE-20 were measured at 134 F g^−1^, 175 F g^−1^, 197 F g^−1^, and 115 F g^−1^, respectively, at a current density of 0.5 A g^−1^ (Figure 5c). It is observed that the specific capacitance of the SSC decreases with increasing current density, a phenomenon attributed to the deterioration of charge transport caused by restricted ion diffusion at the electrode/electrolyte interface under high current density conditions, leading to a reduction in specific capacitance [27]. Nevertheless, the SSC device assembled from LSE-15 maintains a commendable specific capacitance of 120 F g^−1^ even at a high current density of 5 A g^−1^, suggesting its capability to deliver high energy density at elevated charge/discharge rates. The Ragone plots (Figure 5d) indicate that the energy storage capacity of our SSC device exceeds that of previously reported devices [11,28,29]. Specifically, the SSC constructed with LSE-15 achieved a high energy density of 27.33 W h kg^−1^ at a power density of 500 W kg^−1^. Remarkably, even as the power density escalated to 4998 W kg^−1^, the energy density of the SSC remained at 17.22 W h kg^−1^.

As illustrated in the EIS curves (Figure 5e), all observed curves exhibit a semicircular configuration in the high-frequency region, accompanied by a straight line that is nearly perpendicular to the real part in the low-frequency region. This behavior is characteristic of a double-electric-layer supercapacitor, indicating that the SSC composed of binary LNP/SA-based solid electrolyte film and LNP-based LCA electrodes demonstrates favorable double-electric-layer behaviors [30]. In the Nyquist plot from the EIS curves, a reduced radius of the semicircle arc and a straight line slope approaching 90° indicate lower resistance faced by the electrolyte during ion transport, enhanced ion diffusion, and improved electrochemical performance [31]. Notably, the SSC device assembled with the LSE-15 exhibits the lowest Warburg resistance (*R*_s_) of 0.4 Ω and charge transfer resistance (*R*_ct_) of 7.46 Ω (Table 1), indicating a reduced resistance at the electrode/electrolyte interface and high ion diffusion efficiency at the electrode surface [32,33]. This enhanced performance is attributed to the hydrophilic groups present in SA, which increase the hydrophilicity of the film, promote greater electrolyte uptake, and facilitate a more effective film-electrode contact surface.

Cycle performance is a critical factor in assessing the electrochemical stability of the SSC device, evaluated through repeated GCD cycling at a current density of 5 A g^−1^ [31]. After 3000 cycles of constant current charging and discharging, the coulombic efficiency remains at 99%, and the specific capacitance retains 94% of its initial capacity, demonstrating excellent electrochemical reversibility and good cycling stability.

**Figure 6:**
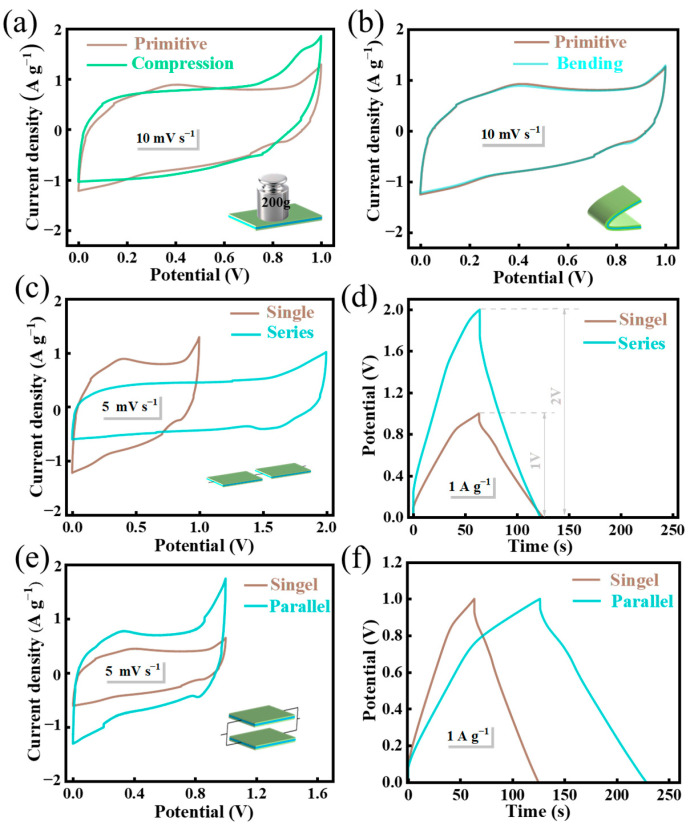
CV curves of the SSC under compression (**a**) and bending (**b**) conditions; the CV (**c**) and GCD (**d**) curves of two SSCs in series; the CV (**e**) and GCD (**f**) curves of two SSCs in parallel.

In order to examine the operational performance of the SSC under complex conditions such as compression, bending, and both series and parallel connections, the CV and GCD performance tests of the LSE-15-based SSC were conducted. As illustrated in Figure 6a,b, the CV curves of the SSC under compressive force (200 g weight pressure) and bending (exceeding 90°) conditions closely align with the original CV curves. This observation indicates that the SSC can sustain normal functionality under both compression and bending conditions, demonstrating commendable stability and significant potential for application in flexible electronic devices. Furthermore, two identical SSCs assembled from LSE-15 were connected in both series and parallel configurations to explore variations in output current and voltage for diverse usage scenarios. The voltage window of the device following series connection is twice that of a single SSC device (Figure 6c,d), which is in accordance with established physical principles [34]. The GCD curve remains in a class-symmetric isosceles triangle shape, indicating that the series connection of SSCs can effectively modulate the voltage window of the device. Conversely, the voltage window of the SSC in parallel configuration remains constant, while the current density is approximately double that of a single SSC device (Figure 6e,f). At a current density of 1 A g^−1^, the SSC in parallel configuration achieves a capacity of 208 F. The highly stable operational performance of the SSC in extreme environments underscores its exceptional potential for practical applications in supercapacitor technology.

## 4. Conclusions

A binary SA/LNP-based solid electrolyte film (LSE) was successfully synthesized by varying the composite ratio of SA and LNPs. The optimal formulation, designated as LSE-15, which contained an SA amount of 15% of LNPs, exhibited the highest porosity (58.4%), liquid/electrolyte absorption capacity (308%), and ionic conductivity (14.10 mS cm^−1^ at 25 °C). This formulation also demonstrated superior electrochemical performance. The SSC assembled using LSE-15 and LCA electrodes achieved a specific capacitance of 197 F g^−1^ at 0.5 A g^−1^. Furthermore, the energy density of the SSC reached 27.33 W h kg^−1^ at a power density of 500 W kg^−1^. Notably, even at a power density of 4998 W kg^−1^, the energy density remained substantial at 17.22 W h kg^−1^. Additionally, the operational stability of this high-performance, all LNP-based SSC device under complex environmental conditions strongly indicates its potential for future applications in energy storage.

## Figures and Tables

**Table 1:** Specific capacitance (*C*), Warburg resistance (*R*_s_) and charge transfer resistance (*R*_ct_) of the SSC.

	*C* (F g^−1^)	*R*_s_ (Ω)	*R*_ct_ (Ω)
LSE-5	134	0.93	18.08
LSE-10	175	0.43	9.89
LSE-15	195	0.40	7.46
LSE-20	115	5.64	25.43

## Data Availability

The original contributions presented in the study are included in the article, further inquiries can be directed to the corresponding author.
